# Presentation of a Case of Short Root Anomaly in an 11-Year-Old Child

**DOI:** 10.1155/2023/1766133

**Published:** 2023-01-04

**Authors:** Angeliki Sofia Trimeridou, Aristidis Arhakis, Konstantinos Arapostathis

**Affiliations:** Department of Pediatric Dentistry, School of Dentistry, Aristotle University of Thessaloniki, Thessaloniki, Greece

## Abstract

Short root anomaly (SRA) is a developmental anomaly in which the affected teeth present morphologically normal crowns and short, round roots. The exact cause of SRA is unknown. A case of an 11-year-old female patient with SRA is described. The patient presented short, round roots of all permanent teeth but first molars. Maxillary lateral incisors presented severe root resorption and mobility. Treatment plan included preservation of the maxillary lateral incisors by splinting them to their adjacent teeth using a stainless steel coaxial wire. A custom mouthguard for dental trauma protection was also constructed using a computer-aided design - computer-aided manufacturing (CAD–CAM) system in order to fabricate 3D-printed dental casts. At 2-year follow-up, the mobility of maxillary lateral incisors decreased, and the patient's dentition remained stable. Considerations regarding differential diagnosis, orthodontic management, and treatment options after an eventual loss of teeth are thoroughly discussed.

## 1. Introduction

Short root anomaly (SRA) is a developmental disorder characterized by permanent teeth with short and blunt roots with closed apices. The affected teeth present a root-to-crown (R : C) ratio of 1 : 1 or less [1]. Permanent central maxillary incisors are almost always affected [1–3], followed by maxillary premolars, maxillary lateral incisors, and mandibular premolars; canines and molars are the least commonly affected teeth [2–4]. SRA teeth are affected bilaterally [1, 4], usually affecting 2–4 pairs of teeth [2]. However, there are rare cases of generalized SRA with multiple teeth affected [4]. The crowns of the affected teeth present normal morphology [2, 4]. Despite the short roots, pulp chambers, root canals, and supporting periodontal tissues appear radiographically normal [1, 2, 4]; however, in some cases, the affected molars may present taurodontism [2, 3]. Clinically, these teeth and periodontal tissues are normal; thus, the condition is often under-diagnosed; it may be diagnosed upon routine radiographic examination or due to tooth mobility [4]. The term hereditary idiopathic root malformation for non-syndromic short roots has also been proposed to describe the condition [4].

Prevalence of SRA varies among the different study populations. Jakobsson and Lind reported that SRA affecting maxillary central incisors was encountered in 2.4% of Swedish children aged 11 years or older [5]. On the other hand, Ando et al. [6] found that 10% of Japanese schoolchildren aged 5–14 years old presented SRA affecting maxillary central incisors. A study on healthy Finnish young adults found a prevalence of SRA of 1.3% [3]. Cutrera et al. [7] reported a prevalence of almost 10% in a sample of 232 patients evaluated for orthodontic treatment; however, the majority of these patients were of Hispanic origin. Moreover, several studies agree that SRA is almost three times more common in females (1 : 2.5–1 : 3.6) [1, 5, 7].

The aim of this study is to report a case of generalized SRA and the management of tooth mobility noticed.

## 2. Case Presentation

An 11-year-and-9-month-old female was referred to our clinic by her orthodontist due to the generalized appearance of short roots in the orthopantomography (OPG).

Patient's medical history was unremarkable; she was born full-term by natural delivery, after an uneventful pregnancy. Parents mentioned recurrent ear infections. Her height and weight were within normal limits for her age, and her blood tests presented no irregular findings. All primary teeth had exfoliated uneventfully, within physiological age intervals, and she had never undergone orthodontic treatment. The patient mentioned a mild dental trauma (probably concussion) at the central maxillary incisors two years earlier, which was not evaluated by a dentist at the time.

At extraoral clinical examination, the patient presented no abnormalities. Upon intraoral clinical examination, permanent incisors, canines, premolars, and first permanent molars were present, and second permanent molars were almost fully erupted. Crowns of all teeth were of physiological size, color, and shape. Oral hygiene of the patient was poor, and the patient presented mild gingivitis. From an orthodontic point of view, she presented an Angle class I molar relationship; the overjet was 2 mm and overbite 3 mm. Mobility of the teeth was evaluated using Miller's classification [8]. Mandibular central incisors presented grade I mobility due to their very short roots, and maxillary lateral incisors presented grade II mobility, due to almost complete root resorption. All maxillary incisors were sensitive to percussion. All upper incisors were sensitive to cold and electrical pulp vitality testing. All lower incisors responded positively to both tests.

**Figure 1:**
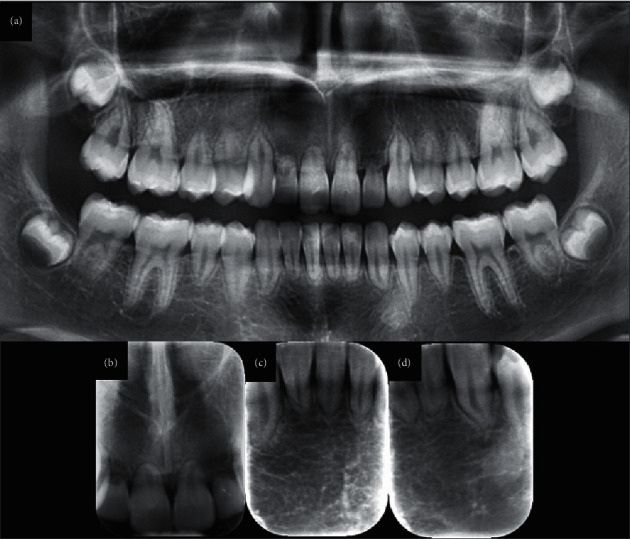
Initial radiographic examination of SRA (age of patient: 11 years and 9 months). (a) Panoramic radiography. (b) Periapical radiograph of the maxillary incisors. (c) and (d) Periapical radiographs of the lower incisors. Notice the abnormally short roots with round apices of all teeth except first molars. Mandibular first premolars also have short roots, but their root to crown ratio is bigger than 1 : 1. Maxillary lateral incisors present intense root resorption and apical radiolucency.

Initial radiographic evaluation included the OPG and periapical X-rays of the incisors (Figures 1(a), 1(b), 1(c), and 1(d)). All teeth except for the first molars presented abnormally short roots with round apices. The mandibular first premolars had also short roots, yet their root to crown ratio was bigger than 1 : 1. The maxillary lateral incisors, however, presented advanced root resorption, with almost no remaining root, and apical radiolucency. Despite their short roots, all other teeth presented normal pulp chambers and root canals. Except for the two maxillary lateral incisors, all teeth presented a physiological lamina dura; no alveolar bone abnormalities were noticed either. Family OPGs evaluation revealed that no other family member (father, mother, or younger female sibling) presented teeth with short roots.

Oral hygiene instructions were given, underlining its importance, especially in maintaining her maxillary incisors as long as possible. The patient was also advised to avoid biting with her anterior teeth.

**Figure 2:**
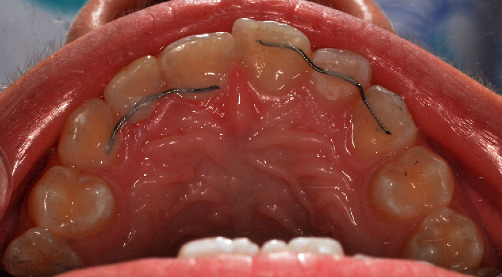
Passive splinting of maxillary lateral incisors to their adjacent teeth. Two separate palatally bonded wires were used.

In order to stabilize the maxillary lateral incisors and prolong their presence in the oral cavity, it was decided to splint them with their adjacent teeth, using a 0.015″stainless steel coaxial wire. The wire was bonded using composite resin at the palatal surface of the anterior maxillary teeth. The placement of a single wire from tooth #13 to tooth #23 would irritate the incisal papilla and interfere in the occlusion; thus, two separate pieces of wire were used to splint the teeth #11–#12–#13 and #21–#22–#23 together, respectively (Figure 2).

**Figure 3:**
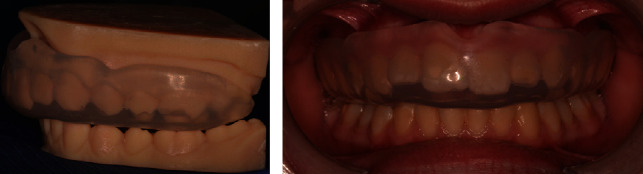
(a) 3D-printed dental casts with the custom-made mouthguard. (b) Intraoral picture of the mouthguard.

The patient played handball at a competitive level, so it was decided to construct a custom mouthguard in order to prevent dental trauma, which would further compromise the maxillary incisors or lead to their loss. Since the roots of multiple teeth were short and the lateral incisors had almost no root, taking dental impressions with a conventional impression material could lead to the accidental extraction of a tooth upon removal of the tray from the oral cavity. Therefore, it was decided to make 3D-printed dental casts instead (Figure 3(a)) using a computer-aided design - computer-aided manufacturing (CAD–CAM) system. The custom-made mouthguard was tried in the mouth (Figure 3(b)); instructions on how to put it on, remove, clean, and store were given. Patient was advised to wear the mouthguard at all times when playing sports.

**Figure 4:**
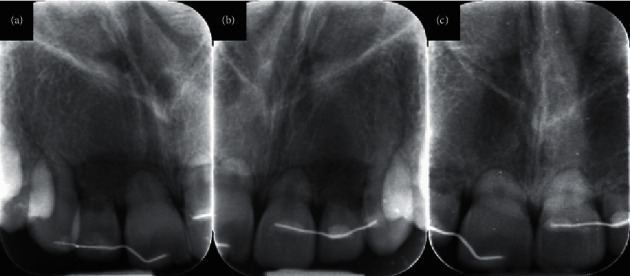
(a) and (b) Periapical radiographs of the upper incisors at 6 months. Slight radiolucent lesions around lateral incisors still persist. (c) Periapical radiograph of the upper incisors at 24 months. Complete healing with no radiolucent area around maxillary lateral incisors.

Close observation, every 3 months, was planned. At 6-months follow-up, the splint was intact, and all maxillary incisors presented no mobility and were no longer sensitive to percussion. However, radiographic reevaluation with periapical radiographs revealed that the radiolucency at the maxillary lateral incisors persisted (Figures 4(a) and 4(b)). Oral hygiene instructions were given again, emphasizing on the palatal surface of the maxillary anterior teeth, where the splint was located. At 24-month follow-up, oral hygiene was good, and all maxillary incisors presented no mobility and no sensitivity to percussion. The 24-month radiographic re-evaluation showed complete healing of the periapical area of the upper lateral incisors and no further root resorption (Figure 4(c)).

## 3. Discussion

SRA is a developmental anomaly of the dental roots, affecting primarily the permanent maxillary central incisors, but other teeth may also be involved, bilaterally. The crowns of the affected teeth appear normal, whereas their roots are short with closed, round apices [1, 2]. The maxillary central incisors present characteristic root morphology with short roots that “lack of significant taper toward the apex” and round apices, whereas mandibular second premolars may appear less round with blunted apices, and thus, they require particular attention not to be misdiagnosed as presenting root resorption [4]. The aetiology of SRA still remains unclear. SRA appears to be the result of an impairment in Hertwigs epithelial root sheath apical proliferation during root development of the affected teeth [9]. Previous studies found an autosomal dominant [2, 4] pattern of inheritance; however, there are also single cases of SRA, which appear to be the result of fresh mutations [2]. Autosomal recessive inheritance has also been suggested [2]. Along with short roots, other dental anomalies have been described in patients with SRA, such as supernumerary teeth [2], tooth agenesis [2, 3], taurodontism [2, 10], peg-shaped lateral incisors [3], microdontia [10, 11], and dens invaginatus [10].

There are several other conditions in which short roots of the teeth may be detected and SRA has to be differentially diagnosed. Children who have undergone radiotherapy of the head-neck region, total body radiotherapy, or chemotherapy for cancer treatment may present short, tapered roots, due to arrested root development and premature apical closure [12–17] or even complete absence of root development [18]. The younger the age of treatment and higher the dose of treatment, the more severe the developmental anomalies of the roots and the teeth in general [16, 17]. Patients with dentin dysplasia type I (DD-I) also present teeth with normal in shape and color crowns or opalescent, amber-colored crowns. Similar to SRA, the roots of the teeth in DD-I are short and blunt; however, the latter present obliterated pulp chambers. Moreover, DD-I teeth may present apical radiolucency in absence of caries. DD-I affects both the primary and permanent dentition [8, 19]. In differential diagnosis, one should also consider molar incisor malformation, a newly introduced dental malformation that affects mainly the permanent molar roots and incisor crowns [20]. Affected molars present characteristically thin, divergent, short hypoplastic roots [20]; pulp chambers appear as narrow slits [21]. The crowns of the affected molars appear clinically normal. Patients may present no symptoms, so the condition is usually discovered after routine radiographic examination. Affected incisors present a characteristic enamel notch at the cervical third of the tooth crown [20]. Finally, differential diagnosis between SRA teeth and teeth with previous trauma depends on their root shape. Teeth with severe trauma history present short roots with a wide open apex and thin radicular walls, as a result of the arrest of radicular development. Short roots have also been encountered in short stature patients [22, 23] and short stature conditions [24, 25]. Short roots of multiple teeth have also been reported in patients with Turner syndrome [26, 27], Frazer syndrome [28], Stevens–Johnson syndrome [29–33], Hallerman–Streiff syndrome [34], and Schimcke immuno-osseous dysplasia [35]. Some metabolic disorders like hypophospatasia [36], pseudohypoparathyroidism [37, 38], and vitamin D-dependent rickets, type I [39] have also been associated with short roots. Spiky-shaped short roots and alveolar bone abnormalities have been reported in patients with thalassemia major [40].

Our case does not fit in any of the above descriptions. First of all, the medical history of the child was uneventful, and blood tests revealed no metabolic irregularity. The crowns of the teeth appear clinically and radiographically normal. Roots of all teeth except for the first permanent molars are short, with closed and round apices. Pulp chambers and root canals do not present obliteration. With the exception of the maxillary lateral incisors presented with extreme root resorption with apical radiolucency and mobility, the supporting periodontal tissues of all other teeth appear clinically and radiographically normal. Based on all these findings, this case was differentially diagnosed as SRA.

In our case, the upper incisors were subjected to minor dental trauma (probably concussion) two years ago. The central incisors presented short roots with closed apices, which means that the cause for the underdeveloped roots was not the trauma they had suffered. The lateral incisors presented severe root resorption and increased mobility. However, the root resorption probably was not due to trauma, since it was symmetrical. Lind [1] mentioned root resorption, especially of the upper incisors, due to embedded canines or, less frequently, due to orthodontic forces or chronic trauma by masticatory forces. Newman [41] also reported root resorption of lateral incisors during the eruption of the canines.

In order to prolong the presence of the maxillary lateral incisors, it was decided to splint the mobile teeth. Both maxillary lateral incisors presented extensive root resorption and periapical alveolar bone loss. Tooth splinting is indicated in this case as an alternative option to extraction and provisional prosthetic therapy [42]. By minimizing tooth mobility, the splint would minimize the forces applied to the remaining tooth root and to the periapical area, promoting the healing of supporting tissues [42]. The aim was to maintain the natural teeth as long as possible. Special attention was given while positioning the splint; it was placed in a way that favors plaque control and keeps from irritating the adjacent soft tissues [42]. Esthetics were of most importance for the patient; therefore, the splint was placed on the palatal surface of the anterior teeth. At 24-month follow-up, mobility was reduced, and the periapical region of the maxillary lateral incisors showed alveolar bone healing with no further root resorption, indicating that splinting was a successful choice of treatment. Other authors have also proposed a splint to stabilize teeth with SRA, especially of incisors with mobility after orthodontic treatment [43–45].

Our patient presented an Angle class I, overjet and overbite within physiological limits, and minor tooth misalignment. However, the application of orthodontic forces in the anterior maxillary region is rather contraindicated in our case [44].

The splint placed on the maxillary anterior teeth may prolong the life span *ο*f the maxillary lateral incisors; however, these teeth have poor prognosis due to their severe root resorption. Depending on the age of the patient at the time, we should consider both provisory and permanent prosthetic solutions. Usually, conservative prosthetic rehabilitation is recommended until patient development ceases [46]. If the lateral incisors are lost before the completion of the patient's development, a Maryland type bridge could be used to restore esthetics. However, it should be considered that a fixed prosthesis is not always recommended in SRA patients, since the adjacent, short-rooted teeth may fail to function as abutments [47, 48]. Alternatively, a removable appliance can be used. Permanent prosthetic rehabilitation with implants is a valid option for SRA patients, since the alveolar bone is not affected. However, the age of the patient should be considered and permanent rehabilitation with dental implants should be postponed until patient development is complete [46].

## Data Availability

All data are available at the Pediatric Dentistry Clinic of the Department of Pediatric Dentistry, School of Dentistry, Aristotle University of Thessaloniki.

## References

[1] Lind V. (1972). Short root anomaly. *Scandinavian Journal of Dental Research*.

[2] Apajalahti S., Arte S., Pirinen S. (1999). Short root anomaly in families and its association with other dental anomalies. *European Journal of Oral Sciences*.

[3] Apajalahti S., Hölttä P., Turtola L., Pirinen S. (2002). Prevalence of short-root anomaly in healthy young adults. *Acta Odontologica Scandinavica*.

[4] Puranik C. P., Chaitanya P., Hill A. (2015). Characterization of short root anomaly in a Mexican cohort – hereditary idiopathic root malformation. *Orthodontics & Craniofacial Research*.

[5] Jakobsson R., Lind V. (1973). Variation in root length of the permanent maxillary central incisor. *Scandinavian Journal of Dental Research*.

[6] Ando S., Kiyokawa K., Nakashima T. (1967). Studies on the consecutive survey of succedaneous and permanent dentition in the Japanese children. 4. Behavior of short-rooted teeth in the upper bilateral central incisors. *The Journal of Nihon University School of Dentistry*.

[7] Cutrera A., Allareddy V., Azami N., Nanda R., Uribe F. (2018). Is short root anomaly (SRA) a risk factor for increased external apical root resorption in orthodontic patients? A retrospective case control study using cone beam computerized tomography. *Orthodontics & Craniofacial Research*.

[8] Miller P. D. (1985). A classification of marginal tissue recession. *The International Journal of Periodontics & Restorative Dentistry*.

[9] Luder H. U. (2015). Malformations of the tooth root in humans. *Frontiers in Physiology*.

[10] Desai R. S., Vanaki S. S., Puranik R. S., Rashmi G. S., Nidawani P. (2006). An unusual combination of idiopathic generalized short-root anomaly associated with microdontia, taurodontia, multiple dens invaginatus, obliterated pulp chambers and infected cyst: a case report. *Journal of Oral Pathology & Medicine*.

[11] Vishwanath M., Chen P. J., Upadhyay M., Yadav S. (2019). Orthodontic management of a patient with short root anomaly and impacted teeth. *American Journal of Orthodontics and Dentofacial Orthopedics*.

[12] Sonis A. L., Tarbell N., Valachovic R. W., Gelber R., Schwenn M., Sallan S. (1990). Dentofacial development in long-term survivors of acute lymphoblastic leukemia: a comparison of three treatment modalities. *Cancer*.

[13] Näsman M., Forsberg C. M., Dahllöf G. (1997). Long-term dental development in children after treatment for malignant disease. *European Journal of Orthodontics*.

[14] Hölttä P., Alaluusua S., Saarinen-Pihkala U. M., Wolf J., Nyström M., Hovi L. (2002). Long-term adverse effects on dentition in children with poor-risk neuroblastoma treated with high-dose chemotherapy and autologous stem cell transplantation with or without total body irradiation. *Bone Marrow Transplantation*.

[15] van der Pas-van Voskuilen I. G., Veerkamp J. S., Raber-Durlacher J. E. (2009). Long-term adverse effects of hematopoietic stem cell transplantation on dental development in children. *Supportive Care in Cancer*.

[16] Nishimura S., Inada H., Sawa Y., Ishikawa H. (2013). Risk factors to cause tooth formation anomalies in chemotherapy of paediatric cancers. *European Journal of Cancer Care*.

[17] Proc P., Szczepańska J., Skiba A., Zubowska M., Fendler W., Młynarski W. (2016). Dental anomalies as late adverse effect among young children treated for cancer. *Cancer Research and Treatment*.

[18] Hernandez M., Pochon C., Chastagner P., Droz D. (2019). Long-term adverse effects of acute myeloid leukemia treatment on odontogenesis in a child. *International Journal of Paediatric Dentistry*.

[19] Ye X., Li K., Liu L. (2015). Dentin dysplasia type I-novel findings in deciduous and permanent teeth. *BMC Oral Health*.

[20] Lee H. S., Kim S. H., Kim S. O. (2014). A new type of dental anomaly: molar-incisor malformation (MIM). *Oral Surgery, Oral Medicine, Oral Pathology, Oral Radiology*.

[21] Witt C. V., Hirt T., Rutz G., Luder H. U. (2014). Root malformation associated with a cervical mineralized diaphragm - a distinct form of tooth abnormality?. *Oral Surgery, Oral Medicine, Oral Pathology, Oral Radiology*.

[22] Gardner D. G., Girgis S. S. (1977). Taurodontism, short roots, and external resorption, associated with short stature and a small head. *Oral Surgery, Oral Medicine, and Oral Pathology*.

[23] Witkop C. P., Jaspers M. T. (1982). Teeth with short, thin, dilacerated roots in patients with short stature: a dominantly inherited trait. *Oral Surgery, Oral Medicine, and Oral Pathology*.

[24] Bazopoulou-Kyrkanidou E., Dacou-Voutetakis C., Nassi H., Tosios K., Kyrkanides S., Damoli M. (1992). Microdontia, hypodontia, short bulbous roots and root canals with strabismus, short stature, and borderline mentality. *Oral Surgery, Oral Medicine, and Oral Pathology*.

[25] Shaw L. (1995). Short root anomaly in a patient with severe short-limbed dwarfism. *International Journal of Paediatric Dentistry*.

[26] Ogiuchi H., Takano K., Tanaka M. (1985). Oro-maxillofacial development in patients with Turner’s syndrome. *Endocrinologia Japonica*.

[27] Pentinpuro R. H., Lähdesmäki R. E., Alvesalo L. J. (2013). Root lengths in the permanent teeth of 45,X females. *Acta Odontologica Scandinavica*.

[28] Kunz F., Kayserili H., Midro A. (2020). Characteristic dental pattern with hypodontia and short roots in Fraser syndrome. *American Journal of Medical Genetics. Part A*.

[29] De Man K. (1979). Abnormal root development, probably due to erythema multiforme (Stevens-Johnson syndrome). *International Journal of Oral Surgery*.

[30] Sangwan A., Saini H. R., Sangwan P., Dahiya P. (2016). Stunted root development: a rare dental complication of Stevens-Johnson syndrome. *Journal of Clinical and Experimental Dentistry*.

[31] Gaultier F., Rochefort J., Landru M. (2009). Severe and unrecognized dental abnormalities after drug-induced epidermal necrolysis. *Archives of Dermatology*.

[32] Bajaj N., Madan N., Rathnam A. (2012). Cessation in root development: ramifications of ‘Stevens-Johnson’ syndrome. *Journal of the Indian Society of Pedodontics and Preventive Dentistry*.

[33] Yongho S., Nanyoung L., Sangho L., Myeongkwan J., Yujin L., Youngmi Y. (2017). Stevens-Johnson syndrome: a case report. *Journal of the Korean Academy of Pediatric Dentistry*.

[34] Robotta P., Schafer E. (2011). Hallermann-Streiff syndrome: case report and literature review. *Quintessence International*.

[35] Gendronneau M., Kérourédan O., Taque S., Sixou J. L., Bonnaure-Mallet M. (2014). Dental abnormalities and preventive oral care in Schimke immuno-osseous dysplasia. *European Archives of Paediatric Dentistry*.

[36] Wei K. W., Xuan K., Liu Y. L. (2010). Clinical, pathological and genetic evaluations of Chinese patients with autosomal-dominant hypophosphatasia. *Archives of Oral Biology*.

[37] Gallacher A. A., Pemberton M. N., Waring D. T. (2018). The dental manifestations and orthodontic implications of hypoparathyroidism in childhood. *Journal of Orthodontics*.

[38] Hejlesen J., Underbjerg L., Gjørup H., Sikjaer T., Rejnmark L., Haubek D. (2019). Dental anomalies and orthodontic characteristics in patients with pseudohypoparathyroidism. *BMC Oral Health*.

[39] Zambrano M., Nikitakis N. G., Sanchez-Quevedo M. C., Sauk J. J., Sedano H., Rivera H. (2003). Oral and dental manifestations of vitamin D-dependent rickets type I: report of a pediatric case. *Oral Surgery, Oral Medicine, Oral Pathology, Oral Radiology, and Endodontics*.

[40] Hazza’a A. M., Al-Jamal G. (2006). Radiographic features of the jaws and teeth in thalassaemia major. *Dento Maxillo Facial Radiology*.

[41] Newman W. G. (1975). Possible etiologic factors in external root resorption. *American Journal of Orthodontics*.

[42] Kathariya R., Devanoorkar A., Golani R., Shetty N., Vallakatla V., Bhat M. Y. (2016). To splint or not to splint: the current status of periodontal splinting. *Journal of the International Academy of Periodontology*.

[43] Marques L. S., Generoso R., Armond M. C., Pazzini C. A. (2010). Short-root anomaly in an orthodontic patient. *American Journal of Orthodontics and Dentofacial Orthopedics*.

[44] Valladares Neto J., Rino Neto J., de Paiva J. B. (2013). Orthodontic movement of teeth with short root anomaly: should it be avoided, faced or ignored?. *Journal of Orthodontics*.

[45] Farret M. M., Farret M. M. (2015). Retreatment of a class II patient with short-root anomaly. *Journal of Clinical Orthodontics*.

[46] Bohner L., Hanisch M., Kleinheinz J., Jung S. (2019). Dental implants in growing patients: a systematic review. *The British Journal of Oral & Maxillofacial Surgery*.

[47] Edwards D. M., Roberts G. J. (1990). Short root anomaly. *British Dental Journal*.

[48] Roinioti T. D., Stefanopoulos P. K. (2007). Short root anomaly associated with Rothmund-Thomson syndrome. *Oral Surgery, Oral Medicine, Oral Pathology, Oral Radiology, and Endodontics*.

